# Hybrid modeling of biological networks: mixing temporal and qualitative biological properties

**DOI:** 10.1186/1752-0509-4-79

**Published:** 2010-06-04

**Authors:** Jonathan Fromentin, Damien Eveillard, Olivier Roux

**Affiliations:** 1 IRCCyN UMR 6597, CNRS & École Centrale de Nantes, Nantes, France; 2 Computational Biology group, LINA UMR 6241, CNRS & Université de Nantes, Nantes, France

## Abstract

**Background:**

Modeling a dynamical biological system is often a difficult task since the a *priori *unknown parameters of such models are not always directly given by the experiments. Despite the lack of experimental quantitative knowledge, one can see a dynamical biological system as (i) the combined evolution tendencies (increase or decrease) of the biological compound concentrations, and: (ii) the temporal features, such as delays between two concentration peaks (i.e. the times when one of the components completes an increase (resp. decrease) phase and starts a decrease (resp. increase) phase).

**Results:**

We propose herein a new hybrid modeling framework that follows such biological assumptions. This hybrid approach deals with both a qualitative structure of the system and a quantitative structure. From a theoretical viewpoint, temporal specifications are expressed as equality or inequality constraints between delay parameters, while the qualitative specifications are expressed as an ordered pattern of the concentrations peaks of the components. Using this new hybrid framework, the temporal specifications of a biological system can be obtained from incomplete experimental data. The model may be processed by a hybrid model-checker (e.g. Phaver) which is able to give some new constraints on the delay parameters (e.g. the delay for a given transition is exactly 5 hours after the later peak of a gene product concentration). Furthermore, by using a constraint solver on the previous results, it becomes possible to get the set of parameters settings which are consistent with given specifications. Such a modeling approach is particularly accurate for modeling oscillatory biological behaviors like those observed in the Drosophila circadian cycles. The achieved results concerning the parameters of this oscillatory system formally confirm the several previous studies made by numerical simulations. Moreover, our analysis makes it possible to propose an automatic investigation of the respective impact of per and tim on the circadian cycle.

**Conclusions:**

A new hybrid technique for an automatic formal analysis of biological systems is developed with a special emphasis on their oscillatory behaviors. It allows the use of incomplete and empirical biological data.

## Background

**Figure 1:**
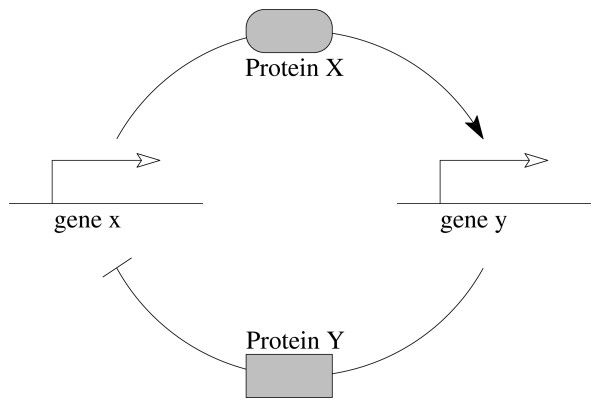
**A two genes interaction network**. Description of a two genes (*x, y*) interaction network. The gene *x *produces the protein X that activates the transcription of genes *y*. It implies a production of the protein Y that represses the transcription of the gene *x*.

Usual experimental approaches studying living systems behaviors focus on various and complementary biological components e.g. a set of genes that encodes a set of proteins. These components interact together within a network. The set of these interactions can be abstracted in a gene regulatory network (GRN), which is the major biological framework for investigating dynamical biological behaviors (see Fig. 1 for illustration). For long, due to the large number of unknown biological parameters (i.e. numerical values of dynamical features related to biochemical reactions), modeling the gene regulatory network behavior was a difficult task. Several approaches try to overcome the lack of parameters values by proposing dedicated qualitative modeling approaches (see [1,2] for overview and [3] for review). They all consider the gene interaction as the cornerstone to represent a biological behavior. It summarizes a protein production that activates or represses the target gene. From a computational viewpoint, these modeling approaches exploit the structure of the network (e.g. interlocked feedback loops) rather than the numerical values of biological compound concentrations. Among the qualitative modeling techniques, approaches based on Piecewise-Affine Differential Equations (PADEs) [4] or the René Thomas's formalism [5] gave astonishing results when applied on concrete biological systems. As shown in [6,7], these techniques correspond to a class of hybrid systems [8] for which we can apply existing powerful techniques for the verification and the control of these hybrid systems. In particular, they permit an automatic investigation of qualitative properties of the genetic regulatory networks [9].

In addition to these modeling features, the last decade saw the emergence of new experimental techniques like micro-arrays [10] that monitor the gene expressions over time. It highlights the recurrent biological interest for biological temporal properties that takes place in all biological scales. Therefore, a new class of hybrid systems, dedicated to biological system modeling, must take into account a new parameter: the time delay. Note that such a parameter was often neglected before, despite documented variations of specific products over time. The time delay represents a unique opportunity to refine existing qualitative models by showing qualitative properties that verify experimental temporal constraints. Conversely, it emphasizes a need for a modeling that includes both qualitative properties, arisen from the biological network structure, and delays associated with the dynamics of genes or gene products. For this purpose, we propose herein a new hybrid modeling technique. We aim at providing a novel tool for the biological community that allows to directly use the qualitative and partial temporal experimental data. Obviously, such modeling does not claim to substitute for existing modelings, but remains a preliminary approach for investigating complex biological system. As a major feature, it abstracts the structure of the network, i.e. positive and negative feedback loops, by focusing on the variation of signs of the gene products following given qualitative behaviors. In this qualitative abstraction, we add some constraints on delays for a natural refinement of the qualitative behavior.

This paper introduces such a hybrid modeling technique. This section highlights connections between our modeling technique and other state-of-the-art modeling approaches, and shows the principle of the modeling. The section *methods *gives a formal description of the hybrid modeling approach, with a special emphasis on qualitative and temporal constraints. The theoretical framework is illustrated on a simplistic system composed of two genes (Fig. 1). Finally, the section *results and discussion *proposes an application of the hybrid modeling on a biological system of reference: the circadian cycle of *Drosophila melanogaster*. This system is particularly well-studied for its temporal properties and hence represents a suitable benchmark for testing our modeling approach and showing reachable biological insights.

### Context and Related Works

Several qualitative modeling approaches, like those using PADEs [4] or discrete abstractions (either boolean abstraction [11] or multivalued abstraction [12]), share similar characteristics but come from different theoretical backgrounds. Discrete abstractions exclusively focus on qualitative data (interlocked feedback loops), which easily lead to determinate parameters values. At the opposite, PADEs systems qualitatively summarize quantitative information to overcome the estimation of parameters that are difficult to obtain. Recently, many works [13-15] demonstrate the promising properties of modeling approaches that incorporate temporal features. Their theoretical frameworks basically use a qualitative modeling that is extended into a hybrid (continuous and discrete) modeling. Among them, Siebert and Bockmayr [15] resume the Thomas's modeling approach [12] and add temporal notions when discrete qualitative parameters are known. It is endowed with a delicate refinement of the discrete dynamics based on temporal parameters. They consider the interval of delays to go from a level *n *to a level *n *± 1 for a given variable. Furthermore, they use timed automation in their modeling which do not allow to consider evolution speeds different from 1. On the one hand, this leads to simple and more efficient model-checking algorithms but, on the other hand, the states graph they get is more complex since they have to deal with distinguished variables standing for either positive or negative or even null evolution rate. Another study proposed by Batt et al. [14] adapts a timed automata approach [16] and extends it from boolean to multivalued discrete states. With their formalism, the authors clearly distinguish the genes and their products. Each gene is represented as a boolean function of all the genes products. The genes products are featured by their concentration discretised levels and their constant evolutions (positive or negative but never null) is a function of their gene (active or not). The action of the gene on the concentration level of its product is delayed according to given delays intervals. Ahmad et al. [13] build models that encompass the consecutive and cumulative increasing and decreasing phases in hybrid automata. Starting from the discrete states graph emerging from the René Thomas approach, they replace each state by a "location" featured by the evolution of all the genes. These authors do not use delays intervals, thus their delays produce deterministic trajectories.

Both timed and hybrid modeling approaches use time intervals in their transitions system, but failed at investigating large networks since the achieved models are quickly too complex for a standard analysis. Our present hybrid modeling technique does not arise from an existing modeling framework. Nevertheless, our model analysis keeps close to the qualitative analysis of a continuous system. For example, there exists a methodology proposed by [17] that derives a qualitative description from ODE systems by a study of the derivative signs. There is also the constraints analysis for large gene regulatory networks proposed by Siegel and co-workers [18]. Their mathematical framework allows to test the compatibility between differential data and knowledge on interactions and then to propose a solution when incompatibility is revealed.

### Principle of our Hybrid Modeling

**Figure 2:**
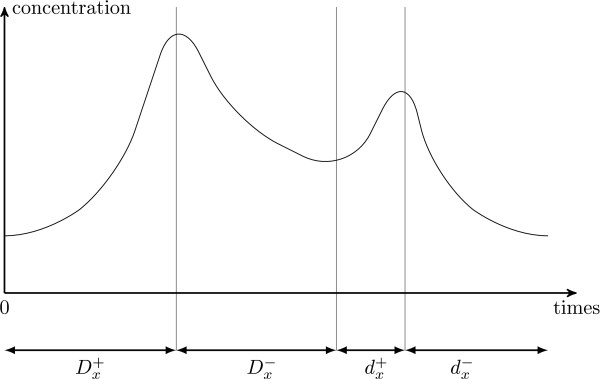
**An example of concentration variation**. Concentration variation of a gene *x*.  (resp. ) represents the maximal decreasing delay of *x *(resp. the minimal decreasing delay of *x*).  (resp. ) represents the maximal increasing delay of *x *(resp. the minimal increasing delay of *x*).

As a major assumption, we consider the *biological qualitative behavior *as the cornerstone of our modeling. By qualitative behavior, we mean the chronological sequence of ordered concentration peaks, rather than their actual concentration values. These peaks have timing properties as well. The knowledge of these properties emerges from experiments but remains often partial. We propose to combine them with the qualitative properties for a better understanding of the system behaviors (see Fig. 2).

**Figure 3:**
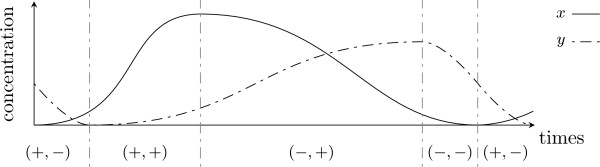
**An example of partition into discrete states**. Concentration variation of the system depicted in Fig. 1 with Y-concentration for dashed curve and X-concentration for other. Such a behavior corresponds to the qualitative cycle (+, -) → (+, +) → (-, +) → (-, -) → (+, -).

Since we focus on the bioproduct peaks, the discrete states, that stand for the time phases separating two such peaks, can be represented by tuples of boolean variables. Each boolean variable - named derivative sign - depicts the behavior of a given gene by showing the increasing time or decreasing time of its protein production. For illustration, in Fig. 3, we have (*x*, *y*) = (+, -) which is, among others, a state standing for an increase of the concentration of the product of *x *(i.e. corresponding to protein X) and a decrease of the concentration of the product of *y *(i.e. protein Y).

Since we are taking into account discrete states for which we are not interested in the actual concentration levels, our hybrid modeling approach does not use the notion of threshold. In the absence of strong assumptions about the interactions of the system, we assume that any interaction can potentially change at any time the derivative sign of the evolution of the target. At this step, the model encompasses behaviors that may not actually take place in the actual executions of the system. They will now be trimmed using temporal constraints. Indeed, our hybrid modeling approach takes into account temporal schedulings, which introduce the notion of time between two successive peaks, since such schedulings rely upon the respective durations of the increase or decrease phases. Thus, it gives some results that are estimations of times to increase and to decrease for each biological variables.

The parameters of our hybrid modeling technique stand for some temporal data in the form of delays, that are not functions of the discrete states. Thus, each transition from one discrete state to another one is defined over a range of delays that build an interval of the possible values of the actual delays. Hence, the transitions between the discrete states are not deterministic. For illustration, in Fig. 2, the increasing time *t*^+^*x *is included in a given interval [, ]. The boundaries of these intervals constitute the set of the temporal parameters values of the hybrid modeling. The number of parameters is therefore a linear function of the number of variables used in the system. Notice that the number of parameters being quite small, it allows to investigate large gene regulatory networks.

Furthermore, by specifying qualitative dynamics, one may obtain parametric results. They correspond to the constraints between the values of the delay parameters of the system. Because the transitions of the hybrid modeling are not deterministic, these parametric results are necessary but not sufficient conditions.

## Results and Discussion

### Implementation and Use

The TEM framework presented in this paper was implemented in a software under the name "GUI-TEM" [Additional file 1: Fig. 2] shows the GUI of this tool) with CeCILL license (French free software license compatible with the GNU GPL) and it is available with its manual on request http://sites.google.com/site/jonathanfromentin/logiciels. The program, written in Java, is multi-platform and provides via its graphic interface, a user friendly tool for analyzing biological models with no specific expertises of the underlying model-checkers (i.e. *HyTech *[19] or *PHAVer *[20]). The protocol to model a given biological system using the TEM approach is the following:

1. Find out the relevant variables of the system (genes and proteins) and their respective interactions.

2. Build the untrimmed TEM via an automatic construction using "GUI-TEM".

3. Provide the known timing specifications (i.e. specific temporal constraints resulting from TEM model such as the structural constraints).

4. Provide the qualitative behavior to be analyzed (i.e. the known chronological sequence of ordered concentration peaks).

5. Analyze and get the results as timing constraints.

### Drosophila Circadian Cycle TEM

**Figure 4:**
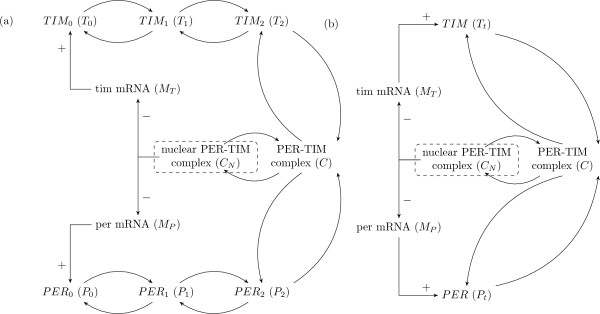
**Interaction and Reaction Systems (IRS) for the Leloup and Goldbeter's model**. Interaction and Reaction Systems for the Leloup and Goldbeter's model where the labeled arrows are interactions (positive (+) or negative (-)) and where the non-labeled arrows are reactions. The **left **part represents the IRS. The **right **part represents the IRS after simplification where *P*_*t *_and *T*_*t *_stand for the total of proteins PER and TIM, as used in our analysis.

The originality of our hybrid modeling approach mainly lies in the use of temporal constraints. Biological models may be separated in two classes. Some models focus on equilibrium behaviors, whereas others point out the oscillatory behaviors of the components. Due to their sensitivity to the parameters estimations, the second class of models tends to be uneasy to analyze. Among them, the most-studied system for its temporal properties is the circadian cycle. A circadian cycle is an oscillation with a period of approximately 24 hours. The complex biological processes underlying this natural rhythm - which takes place in a wide range of organisms - can be summarized by a set of interactions between specific genes. Several models describe the circadian clock of Drosophila cells using Ordinary Differential Equations. Among them, the one proposed by Leloup and Goldbeter [21] shows a particular accuracy with experimental knowledge (i.e. amplitude of oscillations, time series of mRNA and protein concentrations). For all these features, we consider the model of the drosophila circadian clock as an accurate benchmarking for testing our modeling approach. Based on biological assumptions of Leloup and Goldbeter, and following the above protocol, we build the IRS corresponding to the circadian clock model (see Fig. 4(a)). Nevertheless, this model does not exploit in a proper manner the few constraints supplied in the article of Leloup and Goldbeter [21]. Indeed, their biological assumptions mainly deal with the whole concentration of protein PER (*P*_*t*_) and the whole concentration of protein TIM (*T*_*t*_), that are not represented as distinct biological components in the IRS. Since the biological components *PER*_0 _(resp. unphosphorylated protein TIM), *PER*_1 _(resp. monophosphorylated protein TIM) and *PER*_2 _(resp. bisphosphorylated protein TIM) correspond to a simple phosphoric chain reaction that leads to the complexation of proteins PER (resp. TIM), we consider this chain as a single biological compound that abstracts all forms of PER proteins (resp. TIM - see Fig. 4 for details). From these assumptions, we obtain an IRS (depicted in Fig. 4(b)), that leads to a qualitative graph composed of 64 discrete states and 284 discrete transitions (see supplementary materials for details). This model is then automatically analyzed with GUI-TEM.

### Analyzing the Circadian Constraints

By nature, the circadian clock system provides oscillations over a 24 hours period. Hence, we analyzed at first elementary circadian cycle where variables *M*_*P *_and *M*_*T *_are in phase and stand for one high peak and one low peak. This cycle is (+, +, ...) →* (-, -, ...) →* (+, +, ...), with →* describing a finite sequence of discrete transitions (where the first variable is *M*_*P *_and the second variable is *M*_*T *_(other variables are not specified and can take any value)). Furthermore, each discrete transition is related to a set of temporal properties.

Secondly, following the Leloup and Goldbeter assumptions [21], we chose to analyze a period close to 24 hours in conditions of constant darkness. We thus add a clock named *h*_period_, initially null in the discrete state (+, +) and finally at 24 in the same discrete state. We also take into account temporal biological constraints similar to those formulated in the Leloup and Goldbeter study [21]:

• A high peak of *C*_*N *_occurs 5 hours after the high peaks of *M*_*P *_and *M*_*T*_. It implies from a modeling viewpoint, trimming the TEM by adding -- on the guard of the discrete transition corresponding to the high peak of *C*_*N *_-- the conditions *sign*(*M*_*P*_) = - and *sign*(*M*_*T*_) = - (i.e. the later peaks of *M*_*P *_and *M*_*T *_were high peaks), and the conditions  = 5 and  = 5 (i.e. the delay to execute the transition is exactly 5 hours after the later peaks of *M*_*P *_and *M*_*T*_).

• High peaks of *P*_*t *_and *T*_*t *_occur 3 hours after the high peaks of *M*_*P *_and *M*_*T*_. It implies from a modeling viewpoint, trimming again the TEM by adding -- on the guard of the discrete transition corresponding to the high peak of *P*_*t *_or *T*_*t *_-- the conditions *sign*(*M*_*P*_) = - and *sign*(*M*_*T*_) = - (i.e. the later peaks of *M*_*P *_and *M*_*T *_were high peaks), and the conditions  = 3 and  = 3 (i.e. the delay to execute the transition is exactly 3 hours after the later peaks of *M*_*P *_and *M*_*T*_).

**Figure 5:**
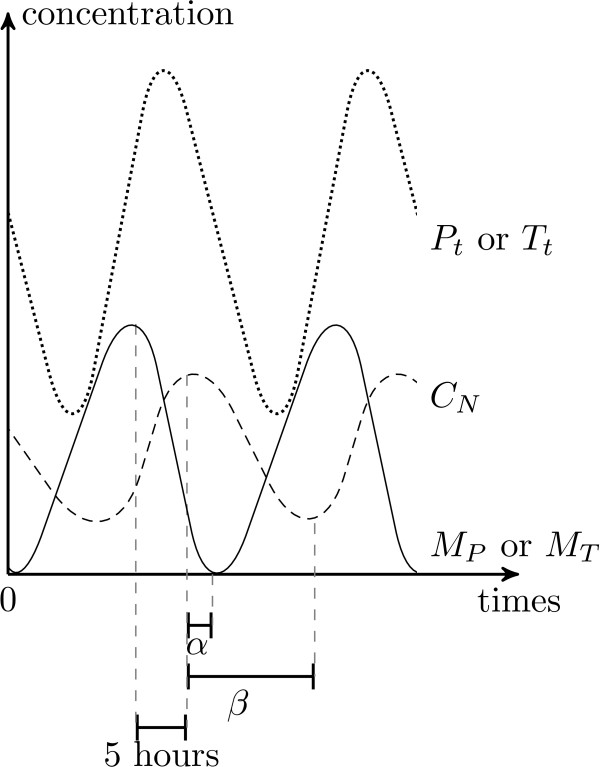
**Gene product concentration variations for the TEM of the rough cycle**. Gene product concentration variations for the TEM of the circadian cycle shown in Fig. 4(b), in accordance with constraints of the rough qualitative cycle. Thus, (i) the peak of CN comes 5 hours after the high peak of *M*_*P *_ahighnd *M*_*T *_and (ii) after the high peak of *C*_*N*_, the decreasing of *M*_*P *_and *M*_*T *_must hold on a delay shorter than the decreasing delay of *C*_*N *_(i.e. *α *<*β*).

These model specifications imply three constraints that are necessary for the existence of such a cycle:

The set of constraints shows several features. First, the constraints (cl) are interpreted as follow: the high peak of *C*_*N *_comes 5 hours after the high peak of *M*_*P *_and *M*_*T*_, so the decreasing of *M*_*P *_and *M*_*T *_must be able to hold on at least 5 hours. In the opposite, the low peaks of *M*_*P *_and *M*_*T *_would come before the high peak of *C*_*N*_. Secondly, the combination of (cl) and (c2) shows that, after the peak of *C*_*N*_, the decreasing of *M*_*P *_and *M*_*T *_must hold on a delay shorter than the decreasing delay of *C*_*N*_. Thus, for an accurate circadian cycle, the specifications of Leloup and Golbeter imply that the low peaks of *M*_*P *_and *M*_*T *_precede the low peak of *C*_*N *_(see Fig. 5). Finally, (c3) clearly exhibits a linkage between the periods of *M*_*P *_and *M*_*T*_. Since, the decrease of *M*_*P *_impacts the period of *M*_*T *_and, conversely, the decrease of *M*_*T *_impacts the period of *M*_*P*_.

**Figure 6:**
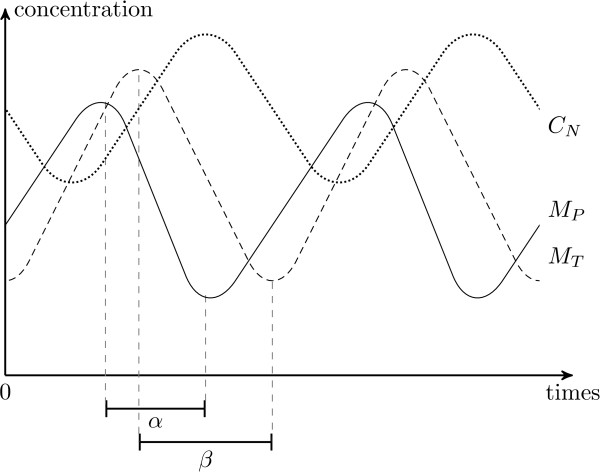
**Gene product concentration variations for the TEM of a specific cycle**. Gene product concentration variations for the TEM of the circadian cycle shown in Fig. 4(b), in accordance with the first qualitative cycle in Table 1. Thus, since the low peak of *M*_*P *_precedes the low peak of *M*_*T*_, then the decreasing delay of *M*_*P *_must be shorter than the decreasing delay of *M*_*T *_(i.e. *α *<*β*).

Beyond this general analysis of the hybrid model, further investigations are possible when one focuses on a cycle of interest like those given in [21]. In this purpose, we indicate the exact occurrence of the concentration peaks of *M*_*P *_and *M*_*T*_. It gives place to four distinct cycles and related constraints, summarized in Table 1, that represent a qualitative variation (i.e. succession of peaks) of the biological products. For each constraints, there exists one disjunction (in the form *A*|*B*, see [Additional file 1]) that emphasizes two distinct regions allowing the existence of the cycles. It means that there are two different sets of possible runs leading to the given dynamical behavior. One of them is less constrained (i.e. the term *A *is less stressed). This particular region is the one that occurs in the larger set of possible runs. It is, hence, potentially more informative. First, Table 1 shows that only the constraints (c3) are different between the four cycles. These new constraints (c3) are stronger (i.e. more restrictive) since  is bounded by  instead of  + , or  bounded by  instead of  + . Secondly, Table 1 shows that only the occurrences order of the low peaks of *M*_*P *_and *M*_*T *_is consequential, since the cycles (1) and (2) give the same constraints (and respectively the cycles (3) and (4)). If the low peak of *M*_*P *_precedes the low peak of *M*_*T*_, then the decreasing delay of *M*_*P *_must be shorter (or identical) than the decreasing delay of *M*_*T *_(see Fig. 6). Similarly, if the low peak of *M*_*T *_precedes the low peak of *M*_*P *_then the decreasing delay of *M*_*T *_must be shorter (or identical) than the decreasing delay of *M*_*P*_. All these constraints are consistent with the simulations obtained from the literature.

**Table 1:** Qualitative cycles of interest where →* describes a sequence of discrete transitions and where the first variable is *M*_*P *_and the second variable is *M*_*T *_(other variables are not specified and can take any value).

Cycle ID	Qualitative Cycle	Necessary constraints
1	(+, +, ...) →* (+, -, ...) →* (-, -, ...) →* (+, -, ...) →* (+, +, ...)	
	
2	(+, +, ...) →* (-, +, ...) →* (-, -, ...) →* (+, -, ...) →* (+, +, ...)	

3	(+, +, ...) →* (+, -, ...) →* (-, -, ...) →* (-, +, ...) →* (+, +, ...)	
	
4	(+, +, ...) →* (-, +, ...) →* (-, -, ...) →* (-, +, ...) →* (+, +, ...)	

### Towards Biological Insights

The previous constraints take place in all simulations and we propose to discuss here their biological meanings. First of all, all above constraints focus on decreasing activities of biological components only. The parameters of greater impact are thus related with the degradations or the repressions. It emphasizes their huge impact on the circadian dynamical behavior. In particular, this result is highly important for setting kinetic parameters of continuous models as modeled by Leloup and Goldbeter [21].

(c1) shows that the longest decreasing delays of per and tim mRNA must not be shorter than five hours.

(c2) indicates that the longest delay to degrade the complex in the nucleus, plus five hours, must not be shorter than the shorter decrease of per and tim mRNA. The combination of both constraints implies a per and tim mRNA productions anterior to the complex production in the nucleus (c.f. Fig. 5). This fact is biologically obvious, but was not part of the initial TEM parameters constraints (i.e. initial biological assumptions). Therefore, it shows an elementary consistency of our model with basic biological knowledge. Furthermore, it indicates that the used biological assumptions are sufficient enough to describe other biological features, that one may call biological artefacts inherent to the model.

Another result concerns the PER TIM complexation. Both proteins form a complex that represses per and tim genes in the nucleus. For theoretical reasons mentioned above, TEM do not abstract such a biological process. The complex compound is hence built using two distinct reactions (i.e. instead of a complexation that must deal with the stoechiometry). Interestingly, TEM analysis exhibits the constraint (c3) that refers to the period of both tim and per mRNA (i.e. sum of delays associated with the increase and the decrease). The tim and per periods are respectively constrained by the shorter decreasing delay for per mRNA and tim mRNA. These constraints clearly state of the impact of PER and TIM on each other: both genes and their products are bound linked by their period, despite the lack of actual complexation in our model. Moreover, it emphasizes that such coupled behaviors are driven by the two negative feedback loops of *C*_*N *_instead of the complex itself.

Each qualitative cycle mentioned in Table 1 implies temporal constraints. The cycles (1) and (2) in this table must satisfy one: the longest decreasing delay of tim mRNA must not be shorter than the shorter decrease of per mRNA. Similarly, the cycles (3) and (4) exist when the longest decreasing delay of per mRNA is not shorter than the shorter decrease of tim mRNA. These constraints relies on the phase synchronicity of the per and tim mRNA. They show that these mRNA degradation rates drive the qualitative scheduling of high peaks over time. For illustration, whatever the scheduling of tim and per mRNA high peaks is, a degradation of tim longer than that of per mRNA implies a low peak of tim mRNA after that of per mRNA (see Fig. 6). Once again, the degradation appears as one of the key factors to control the qualitative oscillatory behaviors.

### Limitations

The major limitation of TEM relies on the limits of the model-checker itself. For practical purposes, the memory space used for the transitions and states recording is high and can not exceed more than a few ten thousand units. Future theoretical developments may overcome these limitations. Pending, we propose practical solutions that reduce the size of the hybrid models:

• Consider only a single delay instead of an interval of delays.

• Consider only the clocks and delays of the relevant variables. Thus, only the discrete dynamics of non-pertinent variables are kept. Tautologies on the guards and the invariants may be used instead of conditions on these delays.

Computational limitations may be overcome by using platforms of computing, as GenoCluster (see the site http://www.genouest.org), that provides distributed reachability algorithms [22].

## Conclusion

We presented here a subclass of Linear Hybrid Automata, named Temporal Evolution Model (TEM). This approach is an accurate first step for modeling living systems with incomplete knowledges. It takes into account (i) a qualitative description of the signs of derivatives, and (ii) the quantitative temporal properties associated with biological productions. These two particular knowledges are notably essential to describe biological behaviors over time, as observed in recent experimental approaches. Thus, based on our hybrid modeling, a qualitative validation of a model consists in finding a peaks scheduling that is consistent with experiments. In addition, TEM provides the opportunity to reason automatically on the temporal properties that are associated with the peaks scheduling. It thus gives a natural refinement of the qualitative validation by showing necessary constraints on delays to achieve a specific qualitative transition, like those observed in the oscillatory behaviors.

In comparison with the other biological hybrid modelings, TEM needs less parameters. The qualitative behaviors are represented only using an interaction system that focuses on the derivative sign variation. This abstraction implies the lost of precise quantitative description (as provided by qualitative thresholds in PADEs), while it allows as well the modeling of larger systems.

We illustrated the potential of our hybrid modeling by the investigation of the Drosophila circadian model. The modeling results are consistent with previous simulations and the literature [21]. These results did not require the parameter settings in a arbitrary way. The investigation of the Drosophila circadian model illustrates the dual perspective that comes from our approach: (i) helping experimental biologists by showing the consequences of their assumptions and (ii) leading modelers to refine their models by trimming unnecessary parameters.

## Methods

### Interaction and Reaction System

We describe a nonlinear dynamical system as being an interaction and reaction system, called IRS, that is defined as follows:

**Definition 1 (Interaction and Reaction System (IRS)) ***An interaction and reaction system (IRS) is a tuple *(*V, I, R*) *where*

• *V is a finite set of biological components*.

• *I *⊂ *V *× α × *V is a finite set of interactions labelled with α *∈ {+, -} *that is the sign of the interaction*. (*υ, α, υ'*) ∈ *I is therefore the interaction of υ on υ', called activation if α *= + *and inhibition otherwise*.

• *R *⊂ *V × V is a finite set of reactions*. (*υ, υ'*) ∈ *R is therefore the reaction of υ moving into υ'*.

Notice that the positive auto-regulations (i.e. interactions in the form (*υ*, +, *υ*)) have no impact on the hybrid model, since such interactions do not change their signs of derivatives. For example, in Fig. 1, the interaction and reaction system is  = (*V, I, R*) such that *V *= {*x*, *y*}, *I *= {(*x*, + *y*), (*y*, -, *x*)} and R = ∅. Notice again that the expressiveness of such a system is not limited by this elementary syntax. For example, a reaction (*υ, υ'*) that requires the presence (respectively the absence) of a component *υ" *not consumed, can be represented by the reaction (*υ, υ'*) and the interaction (*υ", +, υ'*) (respectively (*υ", -, υ'*)). Furthermore, there is at least two way to represent a notion of complexation. The first way represents the complex such as a variable of the system. For example, if the components *υ *and *υ' *form a complex that acts positively on *υ" *then the system provides the reactions (*υ, υυ'*), (*υ', υυ'*) and the interaction (*υυ', +, υ"*). The second (and less precise) way duplicates the interactions and reactions for each component of the complex. Thus, following the example shown above, the reactions of the system become (*υ, υ"*) and (*υ', υ"*).

### Timed Model Design

Based on the previous definition of Interaction and Reaction Systems, we build Temporal Evolution Models (TEM), which are a subclass of the Linear Hybrid Automata (LHA).

Nevertheless, for practical concerns, we will later write TEM systems as hybrid automata, since we want to achieve parametric model-checking analysis (with tools as *HyTech *[19] or *PHAVer *[20]). Up to our knowledge, currently, there are no such tools available for analysing parametric timed automata.

Given a set of variables *X*, let *C*(*X*) be the set of conjunctions of constraints in the form of *x *◇ *c *with *x *∈ *X*, *c *∈ ℚ and ◇ ∈ (≤, =, ≥}.

**Definition 2 (Temporal Evolution Model (TEM)) ***A Temporal Evolution Model (TEM) is a tuple * = *(L, l_0_, H, E, Inv) where*

• *L *= {(*s*_1_,...,*s*_*n*_) | *s*_*i *_∈ {+, -}} *is a finite set of discrete states (discrete states) and n is the number of variables*.

• *l*_0 _∈ *L is the initial discrete state*.

• *H is a finite set of real-valued variables (i.e. the clocks of the system with derivative wrt. time equal to 1)*.

• *E *⊂ *L × C*(*H*) × 2^*H *^× *L is a finite set of edges*. (*1*, *μ*, ℛ, *l'*) ∈ *E is therefore the transition from the discrete state l to the discrete state l', with the guard μ and the set *ℛ *of clocks to be reset upon transition firing*.

• *Inv *∈ *C*(*H*)^*L *^*maps an invariant to each discrete state*.

**Figure 7:**
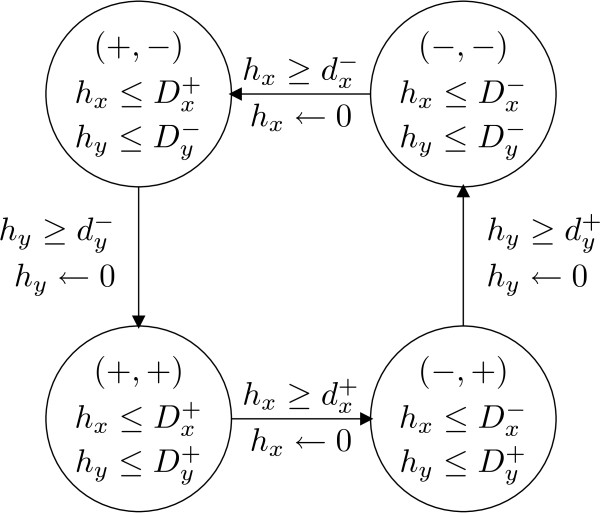
**An example of Temporal Evolution Model (TEM)**. TEM of the network shown in Fig. 1. For each discrete state (like (+, +)), there is a constraint called invariant (*h*_*x *_≤  &*h*_*y *_≤  for (+, +)). For each discrete transition (like (+, +) ← (-, +)), there is both a reset of a clock (*h*_*x *_← 0 for (+, +) ← (-, +)) and a constraint called guard (*h*_*x *_≥  for (+, +) ← (-, +)).

For the running example, we get the following TEM, as represented in Fig. 7.

• *L *= {(+, +), (-, +), (-, -), (+, -)},

• *l*_0 _= (+, -) (arbitrarily chosen, since we focus our modeling application on the oscillatory behaviors, all discrete states are good candidates for a starting discrete state.),

• *H *= {*h*_*x*_, *h*_*y*_},

•  and

• .

The dynamics of the hybrid system are depicted according to both discrete and continuous features that are explained below.

#### Discrete structure (meaning of the discrete states and transitions)

The discrete structure of the TEM is represented by the finite set of discrete states. Let *l *= (*s*_1_,...,*s*_*n*_) be a discrete state with *n *the number of variables and *s*_*i *_∈ {+, -} the sign of the derivative of *x*_*i*_. Thus, for each variable *x*, there are two possible sign values that may be either + (which means that *x *products an activity currently increasing) or - (which means that *x *products an activity currently decreasing), and the cardinality of the set of all the possible discrete states is 2^*n*^. We are mainly interested in the time spent in each discrete state where the evolution of each variable stays unchanged. For example in Fig. 1, the discrete state (+, -) shows that *x *increases while *y *decreases.

The transition from one discrete state to another, is a discrete transition labelled with a guard *μ *such as *h *≥ *p*, where *h *is a clock and *p *a parameter of the hybrid system. A discrete transition stands for a concentration peak of a variable. Thus, the finite set of discrete transitions describes the qualitative dynamics of the system.

#### Continuous structure (chronometric parameterization)

The continuous structure of the TEM is represented by a set of continuous states. A continuous state is defined as a discrete state *l *together with a tuple of real-valued clocks *ν *= (*h*_1_,...,*h*_*n*_). Such clocks evolve with the time. Their evolutions are defined by  and they are constrained by invariants. The clock of a specific discrete state must always verify the invariants of this discrete state. Invariants are conjunctions of constraints, such as *h *≤ *p *where *h *is a clock and *p *is a parameter of the hybrid modeling. For example, the invariant of a discrete state (*s*_1_,..., *s*_*n*_) is

**Figure 8:**
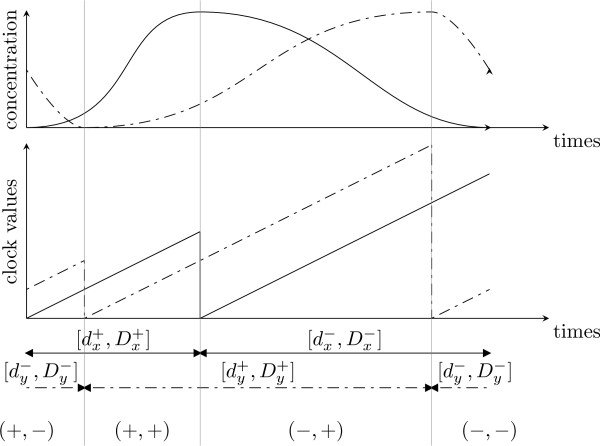
**An example of gene product concentration variations**. Gene product concentration variations for the TEM shown in Fig. 7. The behavior corresponds to the qualitative cycle (+, -) → (+, +) → (-, +) → (-, -) → (+, -). The dashed curves represent the clock and concentration evolution of *Y*. The other curves represent the clock and concentration evolution of *X*. The delay intervals stand for a minimal and a maximal time between two resets of the corresponding clock.

Both guards and invariants are constraining clocks. For example, if the invariant of the discrete state *l *is  and the guard from *l *to *l' *is , then the system stays in *l *during a delay that belongs to the interval [] before it reaches *l'*. Consequently, each variable *x *is associated with 4 parameters that are the boundaries of two delay intervals: [, ] meaning a delay interval where *x *activity increases and, respectively, [] where *x *activity decreases. Fig. 8 shows such parameters with the gene product concentration variations. According to the TEM building, we have, for each variable *x*, the following structural constraint: 0 ≤  ≤  with *α *∈ {+, -}.

#### Building the set of Discrete Transitions from an IRS

At first, we assume that a discrete transition can take place only for at most one switch of variable (at a given time). A discrete transition can exist between two discrete states *l *= (*s*_1_,...,*s*_*n*_) and *l' *= (*s'*_1_,...,*s'*_*n*_) if (∃*j *such that *s*_*j *_≠ *s'*_*j*_. and ∀*k *≠ *j*, *s*_*k *_= *s'*_*k*_). Thus, the rules for building the discrete transitions from the IRS are the following:

• A reaction (*x*_*i*_, *x*_*j*_) such that *s*_*i *_≠ *s*_*j *_implies a discrete transition (*l*, *h*_*j *_≥ , *h*_*j *_← 0, *l'*).

• A reaction (*x*_*i*_, *x*_*j*_) such that *s*_*i *_= + implies a discrete transition (*l*, *h*_*i *_≥ , *h*_*i *_← 0, *l'*).

• An interaction (*x*_*i*_, +, *x*_*j*_) such that *s*_*i *_≠ *s*_*j *_implies a discrete transition (*l*, *h*_*j *_≥ , *h*_*j *_← 0, *l'*).

• An interaction (*x*_*i*_, -, *x*_*j*_) such that *s*_*i *_= *s*_*j *_implies a discrete transition (*l*, *h*_*j *_≥ , *h*_*j *_← 0, *l'*).

### Runs of a TEM

**Definition 3 (Runs of a TEM) ***Any run of a TEM *(*L, l*_0_, *H, E, Inv*) *is an infinite sequence of alternating discrete and timed transitions where*

• *a discrete transition *(*l, ν*) → (*l', ν'*) *takes place if and only if *∃(*l*, *γ*, ℛ, *l'*) ∈ *E such that the guard γ is true for the value ν *(*γ*(*ν*) = *true); we keep the value ν of x, except after a reset (ν'(x) = ν(x) if x ∉ R and 0 otherwise); and the invariant must be true in the target discrete state (Inv*(*l') (ν') = true)*.

• *and a timed transition **takes place with a clock valuation function ν' *= *ν *+ *t if and only if *∀*t' *∉ [0, *t*], *Inv(l)(ν + t) = true*.

For example, let 7 and 12 be the initial values of the clocks *x *and *y*. Thus, ((+, +), (7,12)) is the initial continuous state of the TEM in Fig. 7. After a delay of  - 7, it becomes possible to go in the discrete state (-, +) since the guard (*h*_*x *_≥ ) of the discrete transition ((+, +), (,  + 5)) → ((-, +), (0,  + 5)) is evaluated to true. From this initial continuous state, it is also possible to stay in the discrete state (+, +) during a maximal delay of  - 7. In the discrete state (+, +), the value  of the first clock is the latest delay so that the discrete transition ((+, +), (,  + 5)) → ((-, +), (0,  + 5)) may be fired and the invariant *h*_*x *_≤  be not violated.

### Major Features of the TEM

#### Time between two Concentration Peaks

The minimal (respectively maximal) time between two concentration peaks of the same variable *x *is directly given by the parameters ,  (respectively , ) or a linear expression of these parameters.

Furthermore, the time between a peak *p *of a variable *x *and a peak *p' *of another variable may be given by the clock *h*_*x *_if *h*_*x *_is not reset during this time (because of the occurrence of another peak of *x*). Hence, the temporal constraints, which have to be checked, are in the guard of the discrete transition which coincides with the peak *p'*.

In a most general way, it is possible to use a new clock for the time elapsing between two peaks. The discrete transition, which stands for the peak *p*, has to reset the clock and the guard of the discrete transition, associated with the peak *p'*, contains the temporal constraints.

#### Equilibrium State

By nature, this hybrid modeling is particularly suitable for describing oscillatory dynamics. For this reason, we do not consider null variation signs representing the perfect equilibrium state. The only times where a sign of evolution is null, coincides with a peak of concentration (i.e. a discrete transition). Nonetheless, from the biological point of view, our modeling framework assumes an equilibrium state by the following abstractions:

• an equilibrium state can be viewed as an oscillation with an extremely weak amplitude.

• In one discrete state (*s*_0_,...,*s*_*n*_), a concentration speed can be extremely or asymptotically slow. To deal with such a case, we write ∀*i*,  = +∞. This interpretation is necessary for a modeled system where discrete states without outgoing discrete transition may be reached.

## Authors' contributions

All authors designed the modeling approach and participated in its application. JF implemented the method. JF, DE and OR drafted the manuscript. All authors read and approved the final manuscript.

## Supplementary Material

Additional file 1 **Appendix**. In this additional file, we give all necessary information to obtain the results of the Table 1 with the model-checker PHAVer. Click here for file
