# Supplementary material for: Hybrid modeling of biological networks: mixing temporal and qualitative biological properties
Source: BMC Syst Biol. 2010 Jun 4;4:79. doi: 10.1186/1752-0509-4-79 (PMC2892461; doi:10.1186/1752-0509-4-79)
Supplement: Additional file 1 — Appendix. In this additional file, we give all necessary information to obtain the results of the Table 1 with the model-checker PHAVer. [file 1752-0509-4-79-S1.PDF]

TEM model for the circadian cycle of the drosophila

This section shows the specifications and the results of the TEM model translated for the *PHAVer* tool. In this model, there is no clock for the variable C which does not have temporal constraint. For the notation of specifications and results:

- iMp and iMt allow to select a set of discrete states in function of variables M_P and M_T.
- period is the clock to restrain the cycle at 24 hours.
- loc_010100 is the notation for the discrete state where only M_T and C increase (the order of variables is: M_P, M_T, C_N, C, P_t and finaly T_t).
- The parameters dpMp,DpMp,dnMp and DnMp represent respectively the parameters $d^+_{M_P}$, $D^+_{M_P}$, $d^-_{M_P}$ and $D^-_{M_P}$ in TEM.

Specifications

In this section, we show the command line corresponding to the specifictions of the model which has previously been presented. These specifications are automatically obtained with "GUI-TEM" tool from given path. In Fig. 2, we show its interface for the creation of the model from IRS.

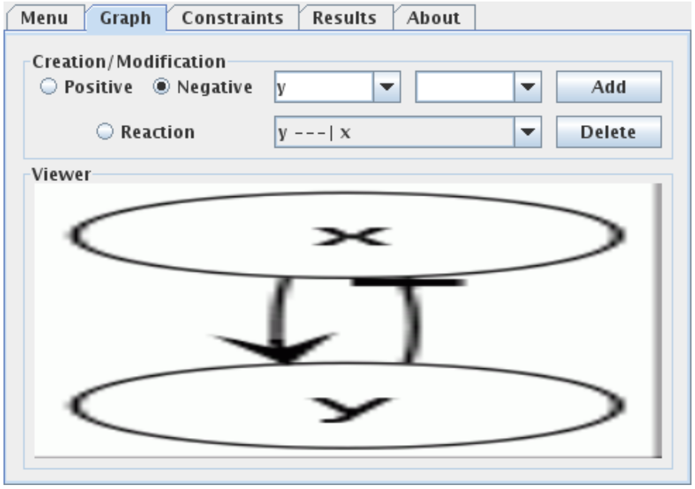

Figure 1: GUI of the TEM. This part allows to create or to change the IRS for tool input. Here, we show the creation of our simplistic system composed of two genes.

Showing these specifications allow to have a better understanding of our results that follow this section. Remark: all phrase after " --" is a commentary.

```
-- The discrete transition maximal number is 6
-- (the number is sufficient for that every variable can bee able to change of state).
REACH_MAX_ITER=6;

-- We use the automata defined previously named GUI.
sys=GUI;

-- Definition of the initial continuous states
-- with initial constraints on the parameters
-- and where mRNA PER and TIM increase (iMp==1 & iMt==1).
initreg_11=sys.{$
& dpMp>0 & DpMp>=dpMp & dnMp>0 & DnMp>=dnMp
& dpMt>0 & DpMt>=dpMt & dnMt>0 & DnMt>=dnMt
& dpCn>0 & DpCn>=dpCn & dnCn>0 & DnCn>=dnCn
& dpP>0 & DpP>=dpP & dnP>0 & DnP>=dnP
& dpT>0 & DpT>=dpT & dnT>0 & DnT>=dnT
& iMp==1 & iMt==1 & period==0};

-- Definitions of the other continuous states for the qualitative cycles.
-- (In the final states the clock for the periode reaches 24 hours).
reg_00=sys.{$ & iMp==0 & iMt==0};
reg_01=sys.{$ & iMp==0 & iMt==1};
reg_10=sys.{$ & iMp==1 & iMt==0};
reg_11=sys.{$ & iMp==1 & iMt==1 & period==24};

-- Specifications for the first qualitative cycle in Table 1.
-- We find all reachable region from initial continuous states.
reg= sys.reachable(initreg_11);
-- We make the intersection
-- with next set of continuous states which we want to reach.
reg.intersection_assign(reg_10);
-- etc.
reg= sys.reachable(reg);
reg.intersection_assign(reg_00);
reg= sys.reachable(reg);
reg.intersection_assign(reg_10);
reg= sys.reachable(reg);
reg.intersection_assign(reg_11);

-- Display the results with projection on the TEM parameters.
reg.project_to(
dpMp,DpMp,dnMp,DnMp,
dpMt,DpMt,dnMt,DnMt,
dpCn,DpCn,dnCn,DnCn,
dpP,DpP,dnP,DnP,dpT,
DpT,dnT,DnT);
```

```
reg_u=reg.loc_union;
reg_u.print;
```

Results

Here, we show the constraints obtained with *PHAVer* for the qualitative cycles of the Table 1 without the redundancy of the initial constraints. Remark: the operator of disjunction is | and the operator of conjunction is &.

- For the cycle number (1):

```
DnCn >= dnMp - 5
& DnMt >= dnMp
& DnCn >= dnMt - 5
& DnMt >= 5
& DnMp >= 5
& DpMp + DnMp >= dnMt
| DpCn + DnCn >= dpMp + dnMp
& DpMt + DnMt >= dpMp + dnMp
& DpCn + DnCn >= dpMp + dnMt
& DpCn + DnCn >= dpMp + 5
& DpMt >= dpMp
& DnCn >= dnMp - 5
& DpMt + DnMt >= dnMp + dpCn - 5
& DpCn + DnCn >= dpMt + dnMt
& DpCn + DnCn >= dpMt + 5
& DnCn >= dnMt - 5
& DpCn >= 5
& DnMt >= 5
& DpMt >= dpCn - 5
& DpMt + DnMt >= dpCn + dnCn
& DpMt + DnMt >= dnCn + 5
& DnMp >= dnMt
& DnMp >= 5
& DnMp + DpMt >= dpCn + dnCn
& DnMp + DpMt >= dnCn + 5
& DpMp + DnCn >= dpMt + dnMt - 5
& DpMp + DnCn >= dpMt
& DpMp >= dpCn - 5
& DpMp + DnMp >= dpMt + dnMt
& DpMp + DnMp >= dpMt + 5
& DpMp + DnMp >= dpCn + dnCn
& DpMp + DnMp >= dnCn + 5
```

- For the cycle number (2):

```
DnCn >= dnMp - 5
& DnMt >= dnMp
& DnCn >= dnMt - 5
& DnMt >= 5
& DnMp >= 5
& DpMp + DnMp >= dnMt
| DpCn + DnCn >= dpMp + dnMp
& DpMt + DnMt >= dpMp + dnMp
& DpCn + DnCn >= dpMp + dnMt
& DpCn + DnCn >= dpMp + 5
& DpMt >= dpMp
& DnCn >= dnMp - 5
& DpMt + DnMt >= dnMp + dpCn - 5
& DpCn + DnCn >= dpMt + dnMt
& DpCn + DnCn >= dpMt + 5
& DpMt >= dpMt
& DnCn >= dnMt - 5
& DnMt >= dnMt
& DpCn >= 5
& DnMt >= 5
& DpMt >= dpCn - 5
& DpMt + DnMt >= dpCn + dnCn
& DpMt + DnMt >= dnCn + 5
& DnMp >= dnMt
& DnMp >= 5
& DnMp + DpMt >= dpCn + dnCn
& DnMp + DpMt >= dnCn + 5
& DpMp + DnCn >= dpMt + dnMt - 5
& DpMp + DnCn >= dpMt
& DpMp >= dpCn - 5
& DpMp + DnMp >= dpMt + dnMt
& DpMp + DnMp >= dpMt + 5
& DpMp + DnMp >= dpCn + dnCn
& DpMp + DnMp >= dnCn + 5
```

- For the cycle number (3):

```
DnCn >= dnMp - 5
& DpMt + DnMt >= dnMp
& DnCn >= dnMt - 5
& DnMt >= 5
& DnMp >= dnMt
& DnMp >= 5
| DpCn + DnCn >= dpMp + dnMp
& DpMt + DnCn >= dpMp + dnMp - 5
& DpMt + DnMt >= dpMp + dnMp
```

```
& DpCn + DnCn >= dpMp + 5
& DpMt + DnCn >= dpMp
& DpMt + DnMt >= dpMp + 5
& DpCn + DnCn >= dnMp + dpMt
& DnCn >= dnMp - 5
& DnMt >= dnMp
& DpCn + DnCn >= dpMt + dnMt
& DpCn + DnCn >= dpMt + 5
& DnCn >= dnMt - 5
& DpCn >= 5
& DnMt >= 5
& DpMt >= dpCn - 5
& DpMt + DnMt >= dpCn + dnCn
& DpMt + DnMt >= dnCn + 5
& DnMp >= 5
& DpMp >= dpMt
& DpMp >= dpCn - 5
& DpMp + DnMt >= dpCn + dnCn
& DpMp + DnMt >= dnCn + 5
& DpMp + DnMp >= dpMt + dnMt
& DpMp + DnMp >= dnMt + dpCn - 5
& DpMp + DnMp >= dpCn + dnCn
& DpMp + DnMp >= dnCn + 5
```

- For the cycle number (4):

```
DnCn >= dnMp - 5
& DpMt + DnMt >= dnMp
& DnCn >= dnMt - 5
& DnMt >= 5
& DnMp >= dnMt
& DnMp >= 5
| DpCn + DnCn >= dpMp + dnMp
& DpMt + DnCn >= dpMp + dnMp - 5
& DpMt + DnMt >= dpMp + dnMp
& DpCn + DnCn >= dpMp + 5
& DpMt + DnCn >= dpMp
& DpMt + DnMt >= dpMp + 5
& DpMp >= dpMp
& DpCn + DnCn >= dnMp + dpMt
& DnCn >= dnMp - 5
& DnMt >= dnMp
& DpCn + DnCn >= dpMt + dnMt
& DpCn + DnCn >= dpMt + 5
& DnCn >= dnMt - 5
& DpCn >= 5
```

```
& DnMt >= 5
& DpMt >= dpCn - 5
& DpMt + DnMt >= dpCn + dnCn
& DpMt + DnMt >= dnCn + 5
& DnMp >= 5
& DpMp >= dpMt
& DpMp >= dpCn - 5
& DpMp + DnMt >= dpCn + dnCn
& DpMp + DnMt >= dnCn + 5
& DpMp + DnMp >= dpMt + dnMt
& DpMp + DnMp >= dnMt + dpCn - 5
& DpMp + DnMp >= dpCn + dnCn
& DpMp + DnMp >= dnCn + 5
```
